# A Retrospective Review of the Use of Regional Citrate Anticoagulation in Continuous Venovenous Hemofiltration for Critically Ill Patients

**DOI:** 10.1155/2013/349512

**Published:** 2013-01-28

**Authors:** Anne Kit-Hung Leung, Hoi-Ping Shum, King-Chung Chan, Stanley Choi-Hung Chan, Kang Yiu Lai, Wing-Wa Yan

**Affiliations:** ^1^Intensive Care Unit, Queen Elizabeth Hospital, 30 Gascoigne Road, Kowloon, Hong Kong; ^2^Department of Intensive Care, Pamela Youde Nethersole Eastern Hospital, 3 Lok Man Road, Chai Wan, Hong Kong

## Abstract

*Background*. The emergence of a commercially prepared citrate solution has revolutionized the use of RCA in the intensive care unit (ICU). The aim of this study was to evaluate the safety profile of a commercially prepared citrate solution. *Method*. Predilution continuous venovenous hemofiltration (CVVH) was performed using Prismocitrate 10/2 at 2500 mL/h and a blood flow rate of 150 mL/min. Calcium chloride solution was infused to maintain ionized calcium within 1.0–1.2 mmol/L. An 8.4% sodium bicarbonate solution was infused separately. Treatment was stopped when the predefined clinical target was reached or the filter clotted. *Result*. 58 sessions of citrate RCA were analyzed. The median circuit lifetime was 26.0 h (interquartile range IQR 21.2–44.3). The percentage of circuits lasting more than 12 h, 24 h, and 48 h was 94.6%, 58.9%, and 16.1%, respectively. There was no incidence of hypernatremia and median pH was <7.5. Hypomagnesemia and hypophosphatemia were detected in 41.6% and 17.6% of blood samples taken, respectively. Although 16 episodes had a total calcium/ionized calcium (total Ca/iCa) >2.5, only four patients had evidence of citrate accumulation. *Conclusion*. The commercially prepared citrate solution could be used safely in critically ill patients who required CVVH with no major adverse events.

## 1. Introduction

Regional citrate anticoagulant (RCA) has been widely used in continuous renal replacement therapy (CRRT) [1–16]. Its use has been greatly simplified by the development of a commercially prepared citrate solution [11–16]. Shum et al. [14] reported a simple citrate regime in 10 critically ill patients using the commercial citrate solution. We would like to expand the use of this regime to a larger group of patients and in an intensive care unit (ICU) with limited prior experience in the use of RCA.

**Figure 1:**
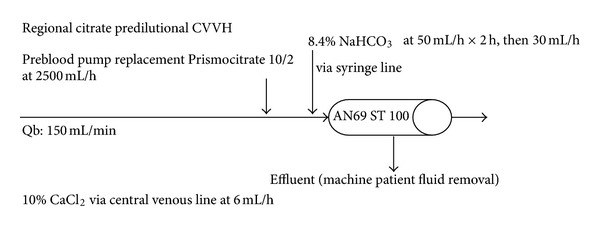
Diagram of predilutional continuous venovenous hemofiltration using Prismocitrate 10/2 solution and Prismaflex machine.

We chose this regime because it is simple to set up. First of all, the blood flow and the substitution fluid rate were fixed to give a constant blood citrate concentration and to eliminate regular measurement of prefilter ionized calcium (iCa). The procedure involved only one replacement solution, Prismocitrate 10/2 (Gambro-Hospal, Stockholm, Sweden). As the amount of base produced was only 30 mmol/L bicarbonate, an external pump was used to infuse the sodium bicarbonate via the heparin port of the circuit to supplement the base required (Figure 1).

Following 3 months of staff training, citrate CRRT was first implemented in our unit on July 1, 2010. The initial response to this regime was suboptimal. This was related to the fact that there was only one dialysis machine that matched this commercialized citrate solution and the unfamiliar use of this new machine by the nursing staff. We subsequently applied this regime to all existing dialysis machines in the unit and the utilization rate rapidly improved.

## 2. Materials and Method

After obtaining approval from our regional Ethics Committee, we conducted a retrospective analysis of ICU patients who underwent citrate CRRT during the period from July to December 2010. Any ICU patients older than 18 years of age who required citrate CRRT for more than 4 hours were included. Indications for starting CRRT included fluid overload unresponsive to diuretic treatment, hyperkalemia with K > 6.5 mmol/L or rapidly rising K, severe acidemia with pH < 7.1, oliguria (urine output <200 mL/12 h), or anuria (urine output <50 mL/12 h). Those with a contraindication for RCA were excluded, including patients with liver disease and bilirubin ≥80 *μ*mol/L and patients requiring massive blood transfusion or high volume hemofiltration ≥3 L/h. All dialyses were performed through a double lumen 12-F hemodialysis catheter (ARROW, Arrow International Inc., USA) inserted into either the femoral or internal jugular vein. Prismocitrate 10/2 (citrate of 10 mmol/L, citric acid 2 mmol/L, sodium 136 mmol/L, and chloride 106 mmol/L) was used as a predilutional replacement solution at a rate of 2500 mL/h. Blood flow was 150 mL/min. A solution of 8.4% sodium bicarbonate was infused at a rate of 50 mL/h for 2 hours, then at 30 mL/h till the end of the treatment. A 10% calcium chloride solution was infused through the central line at a rate of 6 mL/h to replace the loss of the citrate-calcium complex through the circuit. A titration table was used to adjust the rate of CaCl_2_ infusion (5–7 mL/h) to target an ionized Ca of 1.0–1.2 mmol/L. Any negative fluid balance was titrated according to the clinical status of the patient. With the Prismaflex machine, we used the Prismaflex control unit with an AN69 ST 100 hemofilter (Gambro Industries, France). With the MultiFiltrate machine (Fresenius Medical Care, Bad Homburg, Germany), we used the MultiFiltrate HV-CVVHD 600 kit with the Ultraflux AV 600 S hemofilter (Fresenius Medical Care, 1.4 m^2^ surface area, polysulfone membrane).

We monitored electrolytes, arterial blood gas, and iCa every 4 hours during treatment. Total calcium, phosphate, and magnesium levels were measured at least daily. As the median pre- and postfilter iCa in Shum et al.'s work [14] was consistently within the range of effective anticoagulation (0.24 and 0.25 mmol/L), we did not perform the pre-filter iCa check in this study. If the patient had worsening metabolic acidosis that exceeded the previous value by 3–5 mmol/L, the total Ca and anion gap were measured to detect any citrate accumulation. 

Demographic data including age, gender, ideal body weight, APACHE II and IV scores, comorbidity, and reason for RRT support were collected. Circuit effectiveness was measured in terms of circuit lifetime, reasons for stopping CVVH and types of dialysis machine used. Complications related to the use of RCA were defined as metabolic alkalosis with pH > 7.5, hypernatremia with Na ≥ 150 mmol/L, hypokalemia with K ≤ 3.0 mmol/L, hypophosphatemia with PO_4_ ≤ 0.8 mmol/L, hypomagnesemia with Mg ≤ 0.66 mmol/L, low ionized Ca with iCa ≤ 0.85, and total-to-ionized calcium ratio >2.5. Lastly, daily bilirubin level, ICU, and hospital mortality were also recorded.

### 2.1. Statistical Analysis

All continuous variables were compared using Student's *t*-test and the analysis was performed using the Statistical Package for Social Science for Windows, version 16.0 (SPSS, Chicago, IL, USA). The results were displayed as the median with interquartile range (IQR) included. The trend in pH, electrolytes, and base excess was displayed using a standard box plot. This trial was also registered with the Australian and New Zealand Clinical Trials Registry, number ACTRN12611000360910.

## 3. Results

A total of 44 patients received 58 sessions of citrate CVVH. Two sessions were not analyzed as the duration of CRRT was less than 4 hours. The median age was 64 (IQR 57.5–74.5) and the median ideal body weight was 55.5 kg (IQR 51.6–60.0). The median APACHE II score was 29 (IQR 24.8–33) and the APACHE IV was 101.5 (79.3–125.3). Thirty-six patients had preexisting comorbidity and 11 had end-stage renal failure. The three most common reasons for starting RRT were due to sepsis, fluid overload, and after major surgery (Table 1).

**Figure 2:**
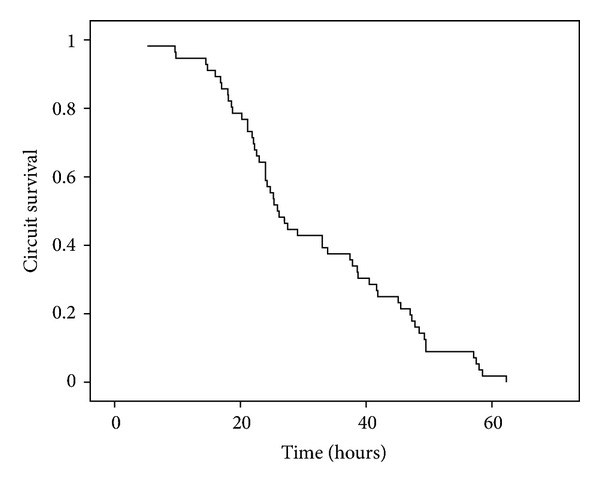
Circuit duration over time.

The median circuit lifetime was 26 h (IQR 21.1–44.3). The maximum duration was up to 62 h. Circuits lasted more than 12 h, 24 h, and 48 h in 94.6%, 58.9%, and 16.1% of cases respectively. Twenty-seven sessions (46.6%) were stopped as the predefined clinical target was reached. Nine were stopped due to circuit failure, three due to cannula problems and four due to citrate accumulation (Figure 2). For circuit with the clotted filter, the median circuit lifetime was 28.0 h (IQR 22.3–44.6 h). Thirty sessions of citrate CVVH were conducted using a Prismaflex machine and 26 sessions with a MultiFiltrate. There was no difference in terms of circuit patency or metabolic control between the two machines.

**Figure 3:**
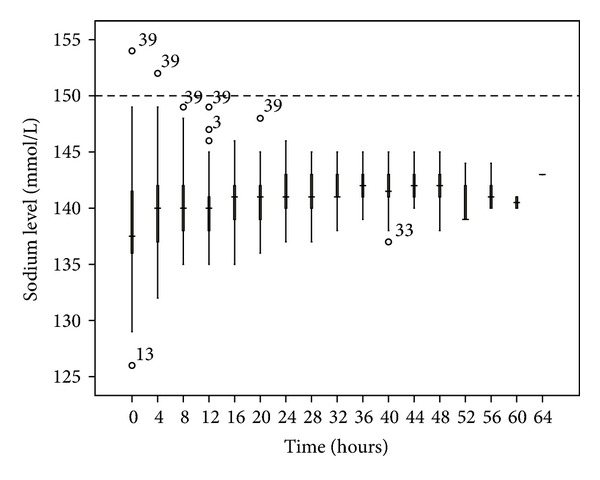
Sodium changes over time during citrate CRRT. Standard box plot in which the horizontal line represents the median, the thick line represents the interquartile range, and the thin line represents the maximum and minimum values. The circular dots represent the outliers.

**Figure 4:**
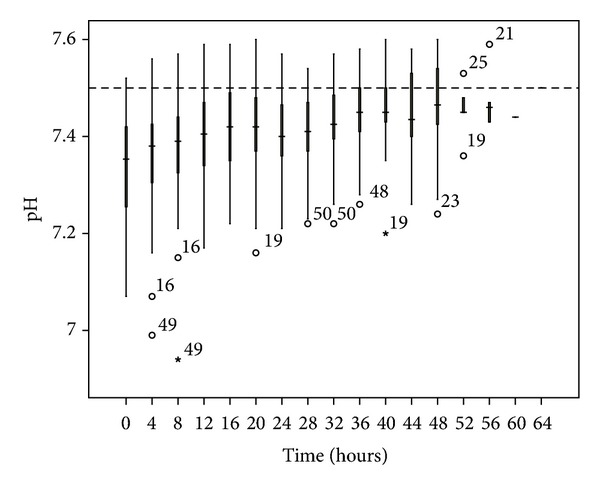
pH changes over time during citrate CRRT. Standard box plot in which the horizontal line represents the median, the thick line represents the interquartile range, and the thin line represents the maximum and minimum values. The circular dots represent the outliers.

**Figure 5:**
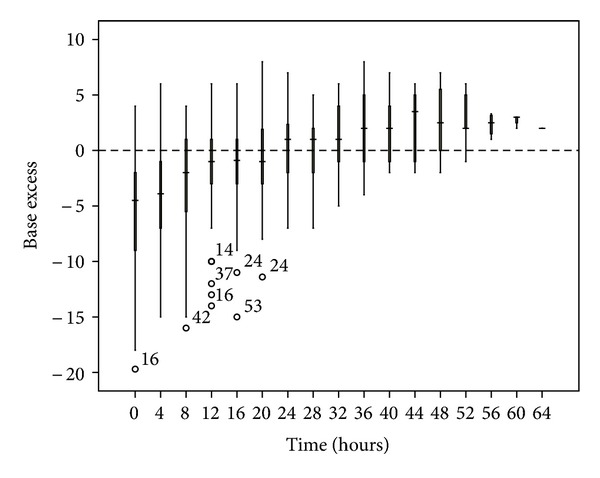
Base excess changes over time during citrate CRRT. Standard box plot in which the horizontal line represents the median, the thick line represents the interquartile range, and the thin line represents the maximum and minimum values. The circular dots represent the outliers.

**Figure 6:**
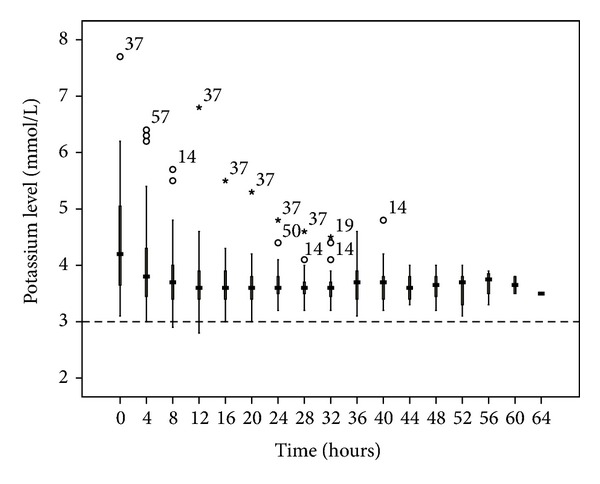
Potassium changes over time during citrate CRRT. Standard box plot in which the horizontal line represents the median, the thick line represents the interquartile range, and the thin line represents the maximum and minimum values. The circular dots represent the outliers.

**Figure 7:**
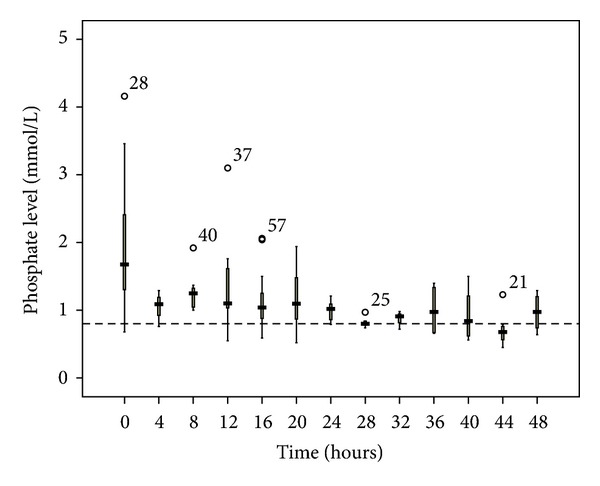
Phosphate changes over time during citrate CRRT. Standard box plot in which the horizontal line represents the median, the thick line represents the interquartile range, and the thin line represents the maximum and minimum values. The circular dots represent the outliers.

**Figure 8:**
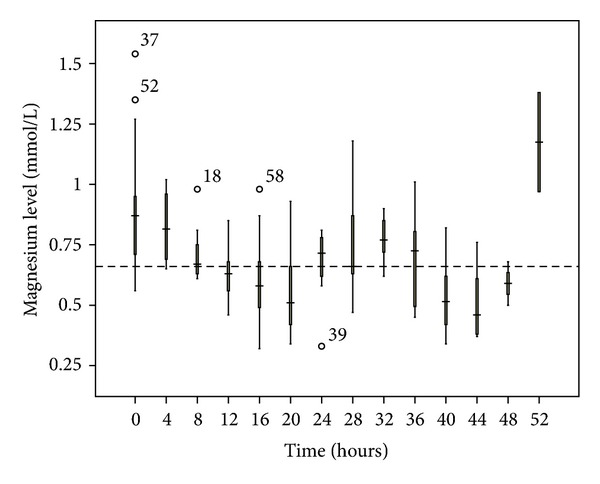
Magnesium changes over time during citrate CRRT. Standard box plot in which the horizontal line represents the median, the thick line represents the interquartile range, and the thin line represents the maximum and minimum values. The circular dots represent the outliers.

No patient developed hypernatremia. The median pH was less than 7.5, although 13.3% of the blood samples had a pH > 7.5. The most common electrolyte disturbance was hypomagnesemia (41.6% of blood samples), followed by hypophosphatemia (17.6%). The time taken to correct the metabolic acidosis as defined by zero-base excess was around 20 to 24 hours (Figures 3, 4, 5, 6, 7, and 8).

**Figure 9:**
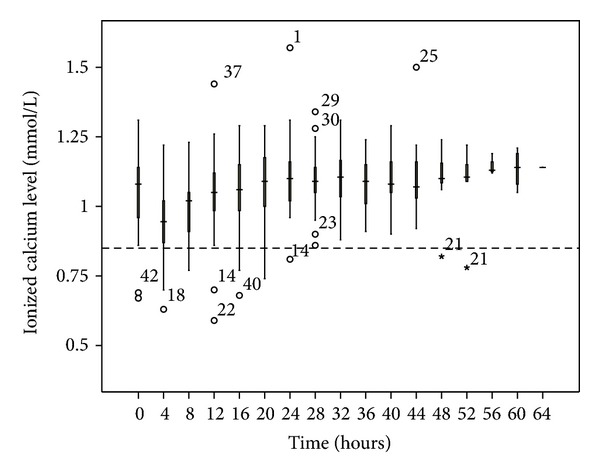
Ionized calcium changes over time during citrate CRRT. Standard box plot in which the horizontal line represents the median, the thick line represents the interquartile range, and the thin line represents the maximum and minimum values. The circular dots represent the outliers.

The median iCa was above 0.85, and only 4.1% of blood samples had values <0.85 (Figure 9). There were 16 episodes of total Ca/iCa > 2.5; but only four patients had CVVH terminated due to citrate accumulation. Three patients had either slow correction or worsening of metabolic control; all had elevated Total Ca/iCa > 2.5 and an increase in anion gap metabolic acidosis. The onset time was 10 to 25 hours after commencement of therapy (Table 2). There were no untoward side effects among these four patients and the metabolic acidosis resolved spontaneously after stopping the citrate CVVH. The ICU mortality rate of this cohort was 23% and the hospital mortality rate was 54.5%.

## 4. Discussion

Various citrate CVVH regimes have been reported, using either trisodium citrate (TSC) [1–7] or Anticoagulant citrate dextrose solution (ACDA) [8–10]. These preparations are usually tailored made, limiting the widespread use of citrate CVVH. Since 2005, commercially prepared citrate solutions have been available on the market and various studies have reported the use of these products [11–16].

Similar to Bihorac and Ross [5] and Schmitz et al.'s studies, [12], Shum et al. [14] used a fixed blood flow to give a constant blood citrate concentration. It has been shown that a blood citrate level of 3–6 mmol/L is required to achieve a systemic ionized calcium of <0.35 [17]. The good anticoagulation efficacy of this regime was demonstrated by the pre- and postfilter iCa of 0.24 and 0.25 mmol/L, respectively [14]. As the citrate is mixed with the replacement solution, we needed to perform predilutional CVVH in our study. As the median body weight in this study was 55.5 Kg, the dose of CVVH was 45.0 mL/Kg/h. Despite the loss of efficiency with predilutional CVVH, it still exceeds the recommended dose of 25–35 mL/Kg/h [18–20].

### 4.1. Citrate Concentration and Circuit Efficacy

The blood citrate concentration of this study was 3.3 mmol/L, which was within the range of 3–6 mmol/L required to achieve effective anticoagulation [17]. The circuit lifetime reported by Shum and another study using a similar preparation in the form of CVVHDF [15] was 50.4 and 58.8 h, respectively. These results compared favorably to the reported circuit lifetime of 48.6–61.5 h [5, 8, 12, 13]. Straaten et al. [21] used a 3-mmol/L blood citrate level and achieved a circuit lifetime of 27 h. Similarly, Brain et al. [16] used a blood citrate level of 2.4 mmol/L and achieved a filter lifetime of 23.6 h. We reported a median circuit lifetime of 26.0 h, which was comparable with the results of these latter two studies. One reason for the shorter circuit lifetime in our study was that we stopped CRRT once the predefined clinical target was reached (e.g., correction of acid-base disturbance or achieving the target negative fluid balance); in contrast, most of the reported studies would run the circuit for 72 hours or until the filter clotted. The circuit failure rate in this study was 16%, which was less than the reported range of 23–49% [1, 5, 8, 12, 21]. Even with a clotted filter, the circuit lasted for a median of 28.0 h (IQR 22.3–444.6). 

### 4.2. Complications Related to the Use of RCA

In terms of metabolic control, we encountered no hypernatremia and only occasional episodes of metabolic alkalosis. This might be related to the use of the exogenous infusion of the sodium bicarbonate. Both hypophosphatemia and hypomagnesemia occurred more frequently in our cohort, highlighting the importance of electrolyte monitoring during the extended period of dialysis after the use of citrate. As the mean treatment dose in this study was higher than the recommended dose (45.0 mL/Kg/h versus 25–30 mL/Kg/h), this might lead to increased loss of the electrolytes through the circuit. Hence, this observation might be a dose-related rather than a citrate-related complication. The addition of 0.75 mmol/L Mg into the substitution solution may help alleviate this problem [13].

In citrate CVVH, a total Ca/iCa of 2.5 has been used as a surrogate of citrate toxicity [22, 23]. The total Ca/iCa was 2.33 in Shum's study [14] and Tolwani et al. [7] reported a maximum total Ca/iCa of 2.8 in a 0.67% TSC group and 2.7 in a 0.5% TSC group. We reported 16 episodes (12.7% blood samples) of total Ca/iCa of >2.5 with four CVVH sessions terminated due to citrate accumulation. Similarly, Morgera et al. [13] reported seven such episodes, four being transient and three persistent. The latter three patients died of hepatic or multiple-organ failure. One interesting finding in our cohort was that the four transiently affected patients had no preexisting liver disease. The onset of citrate accumulation occurs from 10 to 24 hours the after commencement of citrate CVVH. This might be related to the relative hypoperfusion of the liver in critically ill patients with decreased metabolism of citrate and hence a relative accumulation of citrate in the body. All four patients had an increased anion gap and three had either slow correction or worsening of the preexisting metabolic acidosis. None of these four patients had any untoward side effects and the metabolic acidosis resolved spontaneously after stopping the citrate.

A drawback of this study was that it was a retrospective analysis and might, thus, have incurred bias during data correction. In addition, fixing the blood flow and substitution solution rate did not cater for different body weights. It also limited the ability to fine tune the control of metabolic disturbances. Furthermore, an external syringe pump was used to infuse sodium bicarbonate via the heparin port of the circuit to compensate for the inadequate bicarbonate in this regime. As this regime involved the use of the Prismocitrate solution 10/0 only, we preferred to reserve the postfilter pump for another infusion if necessary. The newer models of dialysis machine incorporate software that couples citrate dose and calcium infusion with different blood flows. This advancement will further enhance the control of the dosing of CRRT treatment according to the different needs of patients. Lastly, we had no citrate measurements from patients suspected of citrate accumulation and, thus, were unable to confirm accumulation unequivocally.

## 5. Conclusion

The modified use of this commercially prepared citrate solution in critically ill patients carried a low risk of hypernatremia and metabolic alkalosis. The relatively high incidence of hypophosphatemia and hypomagnesemia might be related to the higher than recommended dosing of RRT given in this study. Lastly, in circumstances in which there is slow correction or worsening of control of metabolic acidosis, the combined use of total Ca/iCa > 2.5 and increased anion gap can help to detect patients with citrate accumulation that may warrant an early termination of RCA.

## Figures and Tables

**Table 1:** Patients' characteristics.

		IRQ (interquartile range)
No. of patients included	44	
No. of treatment episodes	56	
Age (yr) (median)	64.0	57.5–74.5
Gender		
Female	14	
Male	30	
Body weight (Kg) (median)	55.5	51.6–60
APACHE II score (median)	29.0	24.8–33.0
APACHE IV score (median)	101.5	79.3–125.3
No. of patients with comorbidities	36	
DM	25	
HT	30	
IHD	6	
ESRF	11	
Reasons for starting dialysis		
Sepsis	34	
Fluid overload	10	
Major surgery	6	
Prerenal	3	
Others	3	
Baseline blood parameters		
pH (median)	7.35	7.25–7.42
Base excess (median)	−4.5	−9.0 to −2.0
Sodium level (mmol/L, median)	137.5	136–141.8
Potassium level (mmol/L, median)	4.2	3.6–5.1
Phosphate level (mmol/L, median)	1.68	1.30–2.42
Magnesium level (mmol/L, median)	0.87	0.71–0.95
iCa (mmol/L, median)	1.08	0.96–1.14
Total Ca (mmol/L, median)	1.99	1.87–2.10
Urea (umol/L, median)	22.9	17.3–32.2
Creatinine (mmol/L, median)	398.5	284.0–616.8

**Table 2:** Acid-base profile of the four patients with citrate accumulation during citrate CRRT.

	Circuit time (hr)	Base excess changes over time							Anion gap	Total Ca/iCa	Bilirubin (umol/L)
		Baseline BE	4 hrs	8 hrs	12 hrs	16 hrs	20 hrs	24 hrs
Patient 1	9.6	−12	−6	−8	−10				29	4.1	27
Patient 2	24	−3	−5	−3	−3	−5	−4	−1.2	27	2.9	61
Patient 3	9.8	−17	−15	−16					32	2.4	54
Patient 4	16	−14	−11	−11	−13				36	2.5	5
